# Wildfire smoke and health impacts: a narrative review

**DOI:** 10.1016/j.jped.2024.11.006

**Published:** 2024-12-13

**Authors:** Luciana V. Rizzo, Maria Cândida F.V. Rizzo

**Affiliations:** a Universidade de São Paulo, Instituto de Física, Laboratório de Física Atmosférica, São Paulo, Brazil; b Universidade Federal de São Paulo, Departamento de Pediatria – Disciplina de Alergia, Imunologia Clínica e Reumatologia, São Paulo, Brazil

**Keywords:** Air pollution, Biomass burning, Oxidative stress, Cellular damage, Genotoxicity, Epigenetic modulation

## Abstract

**Objective:**

Air pollution emission associated with wildfires is a global concern, contributing to air quality deterioration and severely impacting public health. This narrative review aims to provide an overview of wildfire smoke (WFS) characteristics and associated impacts on adults’ and children's health.

**Data source:**

Literature review based on a bibliographic survey in PubMed (National Library of Medicine, United States), SciELO (Scientific Electronic Library Online), and Google Scholar databases. Observational, cross-sectional, longitudinal, and review studies were considered, prioritizing peer-reviewed articles published in the last 10 years (2014–2024).

**Data synthesis:**

Wildfire smoke (WFS) contributes to the deterioration of air quality, resulting in increased exposure to air pollution especially in wildland-urban interfaces. WFS contains particulate matter (PM) in a range of sizes and chemical compositions, as well as multiple toxic gasses. The health impacts of WFS are systemic, affecting the respiratory, cardiovascular, and neurological systems. Exposure to WFS is associated with inflammatory and oxidative stress, DNA damage, epigenetic modulations, and stress-disorders in adults and children. Children may be at an increased risk of WFS respiratory impacts, due to their smaller airways and developing lungs.

**Conclusion:**

Wildfires are increasing in frequency and intensity, resulting in thousands of premature deaths and hospitalizations worldwide, each year. Preventive measures against wildfire spread must be reinforced, considering the increasing trends of global warming and extreme weather events. Adaptation strategies should be undertaken especially in wildland-urban interface regions, including the improvement of early warning systems, improvement of health care facilities and household preparedness and promotion of risk communication campaigns.

## Introduction

Exposure to WFS is an important health problem, affecting the population in many countries. Wildfires and their associated impacts result from a complex interplay between vegetation type, climate, weather, and human activities. Global climate change is exacerbating wildfire conditions due the increased frequency of heat waves and droughts.^1^ Socioeconomic drivers associated with land use change and agriculture management practices are also important drivers of wildfires. Fire outbreaks associated with deforestation and management of pasture and croplands can easily advance over forest areas under dry weather conditions, amplifying fire emissions. In the Brazilian Amazon forest, deforestation, agricultural management and forest fires respectively account for 8 %, 39 %, and 53 % of the number of active fires.^2^ Extreme wildfire events, characterized by high fire intensity, spread and extension, are increasing in frequency. Between 2016 and 2020, eighteen extreme wildfire events occurred in different parts of the world, including North America, Oceania and South America.^3^

WFS contains a variety of air pollutants, including particulate matter (PM) and trace gases. WFS can be a major contributor to air pollution, particularly in wildland-urban interfaces, increasing public health concerns. Recent studies estimated that wildfires in the United States accounted for 25 % of fine particulate matter (PM_2.5_) concentrations, with 95 % of the population being affected by WFS for at least 1 day per year.^4^

Wildfire-related air pollution has become a major public health concern, as it can be carried out by winds and spread over hundreds of kilometers. Exposure to wildfire emissions is associated with adverse health outcomes, with increased risks of all-cause, respiratory and cardiovascular mortality.^5^^,^^6^ Acute exposure to WFS can exacerbate preexisting conditions, while prolonged exposure can lead to new onset of various diseases.^7^^,^^8^ Respiratory morbidity includes asthma, chronic obstructive pulmonary disease (COPD), bronchitis, and pneumonia. Susceptible populations include people with respiratory and cardiovascular diseases, middle-aged and older adults, children, pregnant women, and fetus. There are relatively few studies about the WFS health impacts in children and that's why this review also includes studies carried out in adults. Considering the lack of evidence of long term adverse health effects from WFS in children, knowledge may be extrapolated from the robust literature on the health effects of air pollutant exposure, especially with the data reported on adults.^9^

This narrative review aims to provide an overview of wildfire air pollutant emissions and associated health impacts worldwide. This review includes a characterization of the air pollutants contained in WFS and their health impacts. Epidemiological evidence of WFS damage to human health is presented, considering global and regional studies. The implications of WFS to oxidative stress, DNA damage, epigenetic modulations, and stress-disorders are summarized in this review.

## Methods

**Figure 1:**
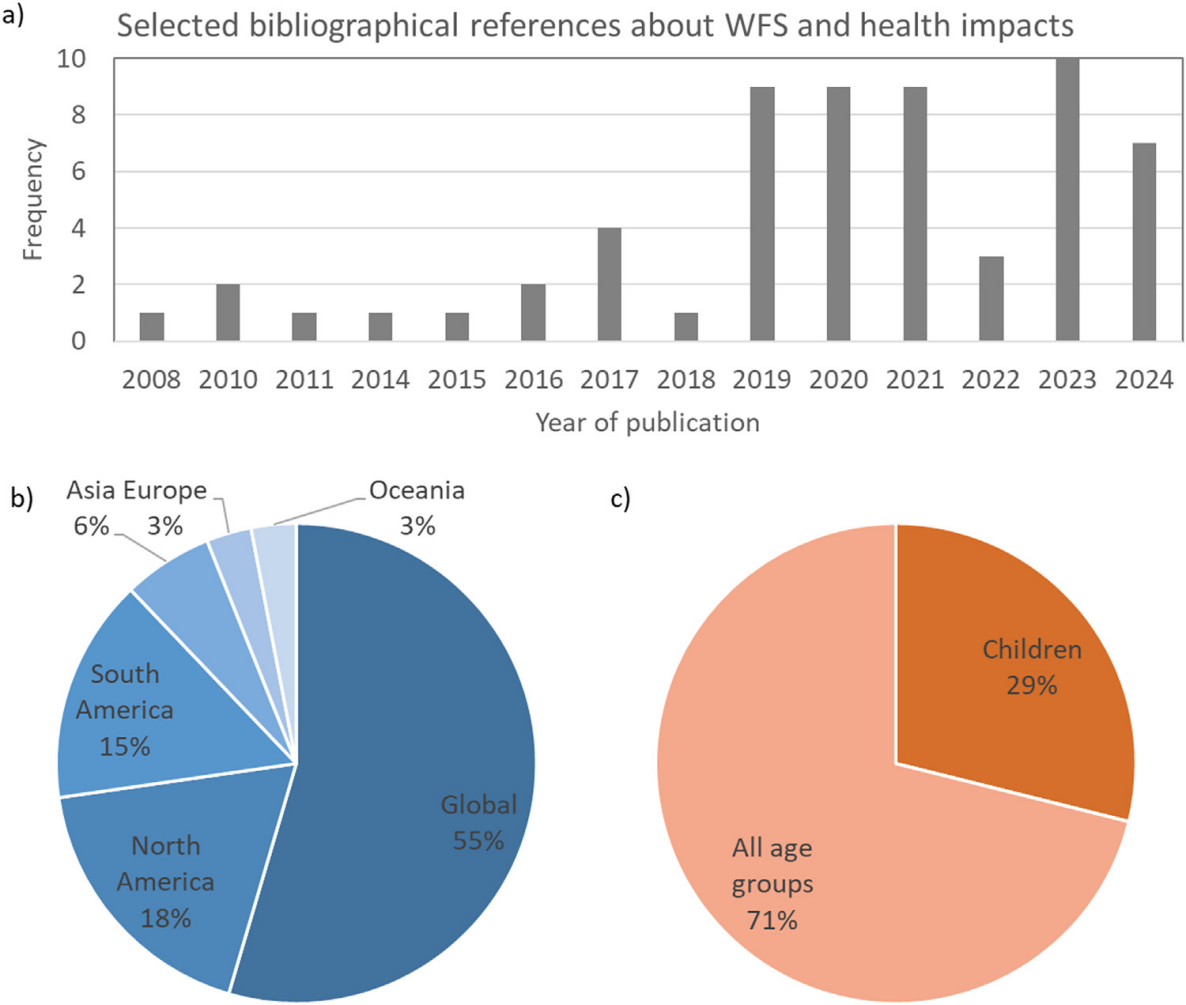
Characterization of the bibliographic references selected for this narrative review. (a) Year of publication; (b) Geographical scope of the findings; (c) Age group focus of health studies.

This is a narrative literature review based on a bibliographic survey in PubMed (National Library of Medicine, United States), SciELO (Scientific Electronic Library Online), and Google Scholar databases. Observational, cross-sectional, longitudinal, and review studies were considered, prioritizing peer-reviewed articles published in the last 10 years (2014–2024). The search was not limited to studies conducted in a particular geographic region. The search terms included wildfire, biomass burning, wildfire health impacts, wildfires and children, wildfires and inflammation, wildfires and oxidative stress, wildfires and genotoxicity, and wildfires and epigenetics. In total, 60 studies were selected, including research (81 %) and review (19 %) articles. 78 % of the studies were published after 2019 (Figure 1a). Concerning the geographical scope of the findings, 55 % of the references had a global scope, while studies conducted in South and North America comprised 33 % of the references (Figure 1a). Most health studies were not focused on a particular age group (71 %), and only 29 % reported findings specifically for children or adolescents (Figure 1c). It reflects the scarcity of studies dedicated to investigating environmental adverse health impacts in children.

### Air pollutants in WFS

Wildfires emit many air pollutants, including particulate matter (PM), nitrogen oxides (NO and NO_2_), carbon monoxide (CO), polycyclic aromatic hydrocarbons (PAHs) and volatile organic compounds (VOC).^10^ The smoke plume is carried away by winds, reaching hundreds of kilometers away from the fire front. Along the transport, the smoke plume suffers dilution, leading to a progressive decrease in the concentrations of primary air pollutants. Chemical reactions transform the air pollutants over time, leading to the production of secondary pollutants like O_3_, secondary organic aerosols, and PAHs.^11^ Recent studies reported increased concentrations of viable airborne bacteria and fungi after biomass-burning events. Fire affects soil microbial community structure and burnt diseased plants might disperse pathogenic spores. Convective air movements associated with fires can launch bacterial cells and fungal spores into the atmosphere. Microbes can also attach to particulates released during combustion.^12^

**Figure 2:**
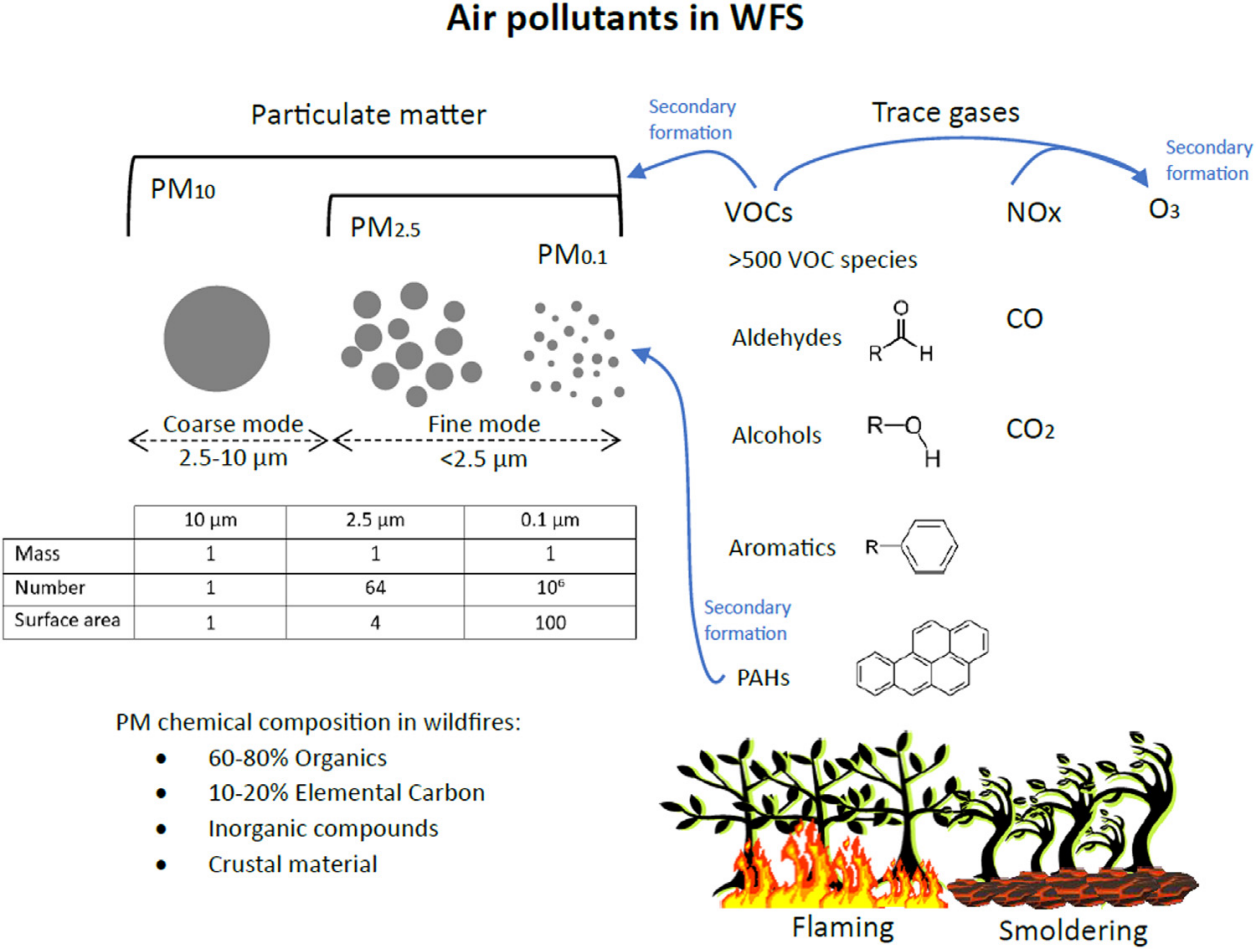
WFS contains a myriad of air pollutants, including particulate matter and trace gases. Photochemical reactions lead to the formation of secondary pollutants like O_3_ and secondary organic PM. The insert table compares the physical properties of three samples of PM with the same mass concentration (arbitrarily defined as 1) but with different particle sizes. Fine mode particles have higher number concentrations and surface area compared to coarse mode particles. The ability of PM_2.5_ and PM_0.1_ to penetrate deeper into the respiratory system and their higher surface area result in higher toxicity compared to larger particles.

Among the air pollutants in WFS, most epidemiological and toxicological studies have focused on particulate matter. PM is not a single toxicant but rather a combination of solid and liquid particles of different sizes and chemical compositions. The physical properties of PM, including mass concentration, surface area, particle number size distribution and physical state, influence respiratory health in different ways. Particulate matter (PM) is categorized by particle diameter: PM_10_, PM_2.5_ and PM_0.1_ refer to particles with diameters below 10 μm (inhalable particles), below 2.5 μm (fine mode particles) and below 0.1 μm (ultrafine particles, UFP), respectively (Figure 2). PM_10_ passes through the upper respiratory tract and enters the proximal and distal airways and alveoli. The smaller the particle diameter, the deeper it can travel into the bronchoalveolar spaces. UFPs have increased toxicity due to their small size and large surface area, being able to enter the alveoli.^13^

Although WFS contains particles of different sizes, PM_2.5_ predominates, comprising >90 % of the particle mass. As the WFS is transported in the atmosphere, physical and chemical processes (oxidation) affect the particle size distribution and the secondary particle formation. Fresh smoke particles typically have mean diameters in the range of 100–150 nm, while aged smoke particles are larger, with mean diameters in the range of 200–300 nm.^14^

Carbonaceous particles, including organic compounds and elemental carbon, comprise 70–90 % of the PM_2.5_ mass in WFS.^15^ PM in WFS may also include crustal elements, heavy metals, PAHs, and inorganic compounds like ammonium sulfate. PM chemical and physical properties depend on the WFS age, fuel, and combustion efficiency. Different stages of combustion (flaming vs. smoldering) produce different species of carbonaceous PM. WFS typically have a long-smoldering phase, sometimes perduring for months, leading to long-term emission of PM_2.5_.^14^

In addition to PM, WFS also contains large amounts of toxic gasses. Urban fires or wildfires in the wildland-urban interface are particularly challenging because the typical WFS components mix with vehicular and industrial emissions, usually accelerating the production of secondary pollutants.^16^ NO and NO_2_ are reactive trace gasses emitted by wildfires and vehicular emissions, known for their oxidant properties and for acting as precursors to secondary air pollutants like O_3_. Exposure to NO_2_ promotes oxidative stress and allergic airway inflammation, increasing asthma susceptibility^17^.

VOCs are organic compounds in the gas phase, and they comprise about 15 % of wildfire emissions by mass.^10^ >500 VOC species have been identified in WFS, having a range of oxygenation states, heteroatoms (e.g., N, F, S), and functional groups (e.g., ketones, carbonyls, alcohols, aromatics).^18^ Polycyclic aromatic hydrocarbons (PAHs) are organic compounds that contain two or more aromatic rings, like benzo-a-pyrene. PAHs can be in the gas phase or in the particulate phase.^19^ The relative abundance of VOC species in WFS depends on the fuel and burning conditions. Typically, 60 % of the VOCs in WFS are oxygenated (e.g., formaldehyde, methanol, acetaldehyde). VOCs undergo oxidation within the WFS generating secondary organic aerosols and O_3_. VOCs are associated with adverse health effects, and the most common diseases associated with VOC exposure are associated with the respiratory system, blood system and inflammation. Acetaldehyde, acrolein, formaldehyde, and benzene are of specific interest because of their differential impact on infants and children compared to adults.^20^^,^^21^

### WFS health impacts: epidemiological evidence in general population

Wildfires contribute to air quality deterioration, especially when it occurs in wildland-urban interface regions, where more people are exposed. Globally, exposure to air pollution contributed to 6.7 million deaths in 2019.^22^ Deaths attributed to PM_2.5_ reached 4.2 million individuals, 57 % from ischemic heart disease and cerebrovascular disease, 36 % from chronic obstructive pulmonary disease and acute lower respiratory tract infections, and 7 % from lung cancer.^23^

Short-term exposure to wildfire-related PM_2·5_ was associated with increased mortality risk by all-cause, cardiovascular, and respiratory diseases at global level, although the magnitudes varied across countries. For each 10 μg/m^3^ increment in wildfire-PM_2.5_, meta-analysis results presented a pooled relative risk (RR) of 1.019 (95 % CI 1.016–1.022) for all-cause mortality, 1.017 (1.012–1.021) for cardiovascular mortality, and 1.019 (1.013–1.025) for respiratory mortality.^5^ In Brazil, respiratory deaths linked to daily exposure to fine particulate matter from wildfires totaled 31,287 from 2000 to 2016.^24^ Specifically in the outstanding fire season of 2019, Nawaz and Henze^25^ estimated 5,0 (2,4–8,3) thousand premature deaths attributable to wildfire-related PM_2·5_ in Brazil. During the Australian 2019–20 megafires, Johnston^26^ estimated 429 smoke-related premature deaths, 3230 hospital admissions for cardiovascular and respiratory disorders and 1523 emergency attendances for asthma.

The health impacts of air pollutants can manifest in any compartment of the human body, including the respiratory system, the immune system, the skin tissues, the sensory system, the central and peripheral nervous system, and the cardiovascular system. The age groups of 0–4 years and 60 years and above exhibit heightened vulnerability to wildfire-related respiratory issues as well as individuals of lower socioeconomic status and pregnant women, for various reasons.^21^^,^^24^^,^^27^ Individuals in the age group above 60 years are more prone to a weakened immune system, declined lung function, and pre-existing health conditions. The risk groups may also have limited mobility or encounter challenges in adopting protective measures during a wildfire event, increasing their vulnerability.^24^

## WFS impacts on pediatric health

Young children, with developing immune systems and smaller airway sizes, are more susceptible to respiratory irritants associated with WFS. Children can be more exposed to WFS because they usually spend more time outdoors. In addition, children breathe more air relative to their body weight, making this group especially vulnerable to air pollution.^9^ Children have less efficient nasal filtering compared to adults, indicating that a higher proportion of PM can penetrate into the lungs.^28^ Moreover, the effects of air pollution in the developing lungs in utero and in childhood may influence adult lung structure and function. Depending on the timing of exposure, some effects can persist or become apparent after many years.^29^ This is a critical point that must be considered: the long-term health effects of wildfire smoke exposure in pediatric populations are currently not known.^9^ Evidence from an observational study on non-human primates suggested that WFS exposure during infancy results in attenuation of innate immune system responses and reduced lung volume in adolescence.^30^

WFS exposure was consistently associated with enhanced risks of adverse health outcomes in children and adolescents, except for cardiovascular morbidities. A recent systematic review of the respiratory risks of WFS exposure in children selected 59 studies from 14 countries.^31^ They reported a significant increase in respiratory emergency department visits and asthma hospitalizations within the first 3 days of exposure to wildfire smoke, particularly in children < 5 years old. A nationwide time-series study in Brazil reported a 4.88 % risk increase of all-cause hospitalization in children ≤ 4 years old per 10 μg/m^3^ increase in wildfire-PM_2.5_ concentrations.^32^ In California, a study has shown that wildfire-PM_2.5_ was 10 times more harmful to children's respiratory health than PM_2.5_ from other sources, particularly for children aged 0 to 5 years^33^.

In addition to the physical health impacts of the WFS exposition, it is important to highlight the detrimental effects of wildfire disasters on mental health, especially for children and adolescents. A recent review identified several stress-related effects after wildfire events, such as post-traumatic stress disorder, anxiety, depression, stress and hopelessness.^34^ A wildfire event in a child's community may be traumatic, so children may exhibit responses such as separation anxiety, disturbances in sleep patterns, increased bedwetting, and trauma re-enactments. A study conducted 18 months after a large wildfire in Canada, that resulted in the evacuation of an entire city, has shown a 31 % prevalence of major depression disorder and suicidal ideations in students impacted by the wildfire, in addition to 27 % probable anxiety and 15 % probable alcohol/substance.^35^

## WFS: inflammatory and oxidative stress

Human exposure to PM is associated with multiple respiratory diseases including, COPD, asthma, interstitial lung damage, and lung cancers.^36^ PM_2.5_ hinders mucociliary clearance with reduced ciliary beat and consequent mucus hypersecretion. In addition to blocking the airways, there is greater microbial proliferation that further induces tissue inflammation.^37^ Systemic effects of WFS exposure may occur via several potential mechanisms: PM interacting with neural receptors in the lungs and activating the autonomic nervous system; a local and systemic immune response to pollutants in the alveoli; and direct translocation of UFP across the alveolar membrane resulting in systemic endothelial dysfunction, activation, and injury.^37^ The oxidative effects of PM can damage mitochondria, endoplasmic reticulum and DNA, can be carcinogenic and activate cell death signaling pathways.^36^

**Figure 3:**
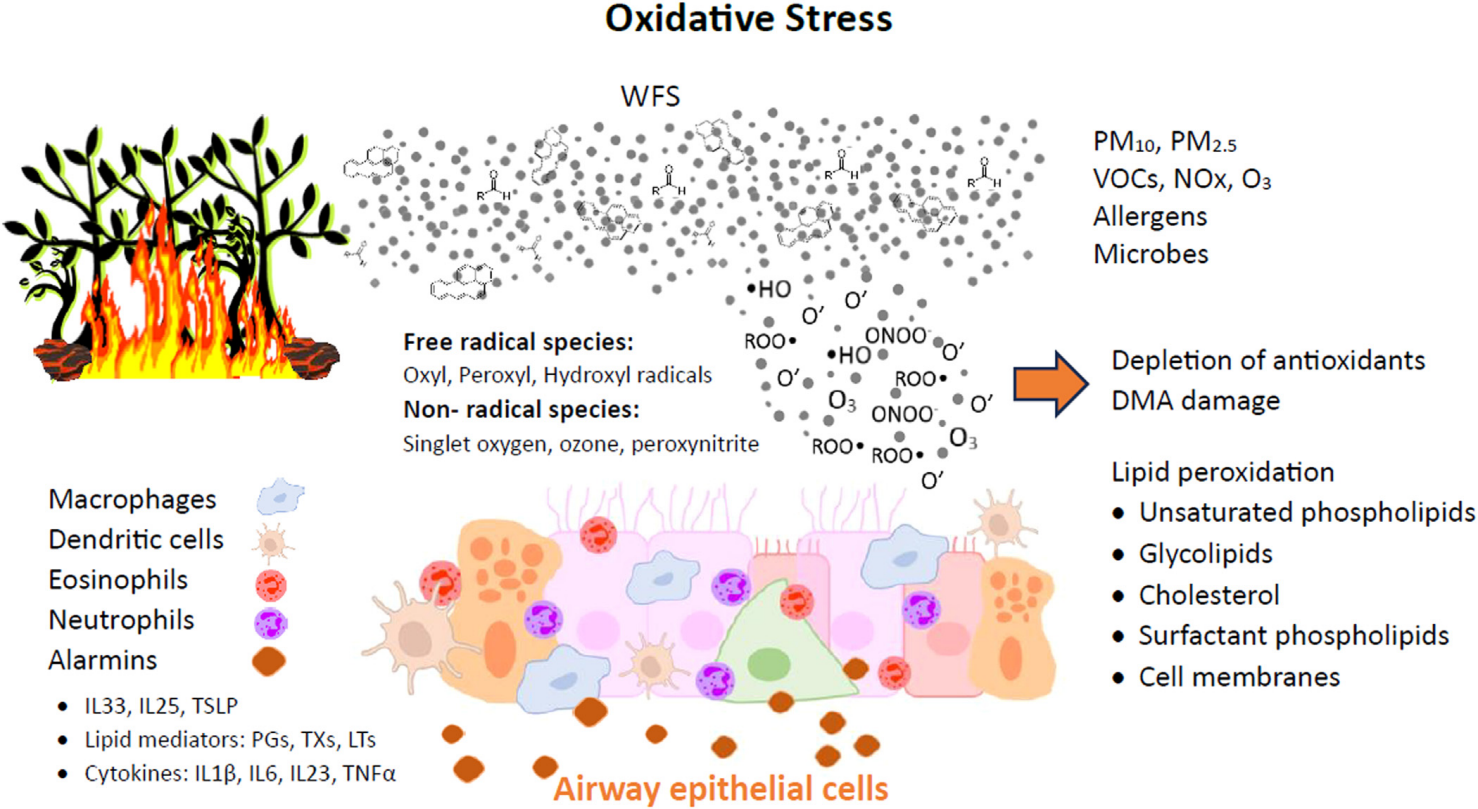
Oxidative stress induced by wild fire smoke leads to damages in the airway epithelial cells. Alarmins are proteins released by cells in response to injures, initiating the inflammatory response, including: IL (interleukin); TSLP (thymic stromal lymphopoietin); PGs (prostaglandins); TXs (thromboxanes); LTs (leukotrienes); TNF (tumor necrosis factor). Adapted from Bowman et al.^21^

The mechanisms of WFS effects on the lower airways and lungs, under asthmatic or healthy conditions, are likely complex. The disruption of the epithelial barrier by oxidative stress in the airways can permit the penetration of pathogens, allergens, and other toxins into the underlying submucosal tissues. Cytokines (IL-1β, IL-6, IL-23,TNF-α) and other inflammatory mediators such as arachidonic acid derivatives and damage-associated molecular patterns (DAMPs) are also released, giving rise to a cascade of proinflammatory changes in structural and immune cells in the respiratory mucosal tissue (Figure 3).^38^^,^^39^

The inflammasome, which is a component of the innate immune system, is a multiprotein complex that regulates inflammation by activating proinflammatory cytokines. The inflammasome activates the pro-caspase-1, which cleaves pro interleukin- 1β, causing greater severity of inflammation.^40^ Studies have shown that exposure to UFP augmented the expression of key components of the inflammasome, such as NLRP3 and caspase-1, leading to the production of active caspase-1 in the lung.^41^ Exposure to PM_2.5_ can also activate cytokine-dependent autophagy pathways.^36^

Another plausible pathophysiological mechanism of airborne particles on human health is oxidative stress (OS). OS is highly implicated in the pathogenesis of respiratory diseases (asthmatic airway changes) and is caused by endogenous overproduction of free radical species as reactive oxygen species (ROS), such as superoxide (O_2_^−^), hydrogen peroxide (H_2_O_2_), and hydroxyl radical (OH).^21^ Cellular organelles such as mitochondria and peroxisomes are major sources of ROS and nitrogen species (NOx).^40^ Oxidative stress is closely related to inflammation in a broad conception.

ROS influences airway cells reproducing asthma pathophysiological features. ROS are associated with acute and chronic lung inflammation inducing lipid peroxidation, protein structure alteration, increased release of arachidonic acid from cell membranes, and increased synthesis of chemoattractants. The release of tachykinins and neurokinins augments airway smooth muscle contraction, increases airway reactivity and airway secretions, increases vascular permeability, decreases cholinesterase and neutral endopeptidase activities, and impairs the responsiveness of β-adrenergic receptors.^40^

The pathophysiology of the asthmatic reaction triggered by allergen stimulation starts with a sensitization phase, in which the allergen contact with naïve T cells results in the production of Th2 cytokines (interleukin (IL)−4 and IL-13) and activation of allergen-specific IgE-producing plasma cells. These IgE antibodies bind to the high IgE affinity receptors on mast cells and basophils awaiting a later allergen exposure. Concomitantly, epithelial injury leads to the activation of innate lymphoid cells type 2 (ILC2) and consequent release of pro-type 2 cytokines IL-33, Thymic Stromal Lymphopoietin (TSLP) and IL-25, known as alarmins. The alarmins contribute to increasing stimulation of DCs in antigenic recognition and ILC2. The effector phase of the asthmatic reaction following a second allergen contact, involves bronchial smooth muscle cell contraction, activation of ILC2 and Th2 cells and eosinophil recruitment.^42^

The cell response to ROS often involves the activation of intracellular signaling pathways and transcriptional changes, which are influenced by the redox status.^43^ Redox status in the nucleus affects histone acetylation and deacetylation processes, regulating the expression of inflammatory genes by activation of transcription factors such as nuclear factor-κB (NF-kB) and activation protein-1 (AP-1). NF-κB is a protein heterodimer that is activated in epithelial cells and inflammatory cells during OS, leading to the upregulation of many proinflammatory genes,^44^ related to the pathogenesis of asthma. AP-1 is a protein dimer composed of a heterodimer of Fos and Jun proteins, regulating inflammatory and immune genes in oxidant-mediated diseases.^40^

Another transcription factor highly expressed in the lungs of humans is Aryl hydrocarbon Receptor (AhR), which can be activated by exposure to PAHs. AhR is a ligand-activated transcription factor that has been shown to play an important role in asthma control.^45^ AhR may have beneficial and deleterious roles. On one hand, AhR activation in dendritic cells (DC) blocks the generation of pro-inflammatory T cells. AhR expression by Treg, acts as an important negative regulator of the allergic reaction. On the other hand, AhR activation by PAHs is pro-inflammatory, inducing mucus hypersecretion, airway remodeling, dysregulation of antigen cells and exacerbating asthma features^45^. Epithelial cells, mast cells, macrophages, B lymphocytes, Th17 cells and potentially ILC2 express AhR. In response to activation by a ligand, AhR translocates from the cytoplasm to the nucleus where it controls the transcription of a variety of target genes with the production of many pro and anti-inflammatory cytokines. AhR also interacts with other transcription factors like NF-kB, modulating their activity and the expression of their target genes.^46^

In non-T2 mediated forms of asthma, such as WFS-induced asthma, recruitment of neutrophils is intensified by the activation of Th17 cells and by the release of CXCL by epithelial cells.^47^ The influx of neutrophils in asthma driven by WFS oxidative stress results in an important clinical problem, since neutrophils are poorly responsive to glucocorticoids,^38^^,^^48^ which is the main therapy indicated for the treatment of asthma and other chronic inflammatory diseases.

Although many of the adverse effects described here refer to PM_2.5_ exposure in general, there is observational evidence that wildfire-specific PM_2.5_ is more harmful to human health than urban PM_2.5_.^33^^,^^49^ Activation of cytochrome P450 genes, a family of enzymes involved in many biological and pathological processes, was more intense in WFS-PM compared to urban-PM. On the other hand, urban-PM was associated with greater levels of the proinflammatory cytokines. Differences in the PAH composition in urban and WFS PM may explain the increased cellular oxidative damage by exposition to wildfires.^49^

## WFS -Genotoxicity and epigenetic modifications

The mixture of various compounds present in WFS significantly influences the toxicity of air pollutants, due to the activation of multiple pathways during exposure. Genotoxicity induction by air pollutants results in the activation of DNA repair mechanisms. However, there is evidence that PAHs and heavy metals could weaken the repair mechanisms.^50^ Mice exposed to high PAHs concentrations in PM_2.5_ presented deficiencies in the DNA repair system, contributing to the development of lung cancer.^51^ Heavy metals induce genotoxicity and epigenetic modifications through a variety of mechanisms, like activation of oxidative DNA damage and altering stabilization of the chromatin.^50^

Epigenetics generally refers to gene regulations that are not associated with alteration in DNA sequence (mutation). Epigenetic mechanisms can alter genome expression under exogenous influence and guarantee the propagation of gene activity states through subsequent cell generations. Alterations in epigenetic marks have been associated with diseases like cancer, cardiovascular, respiratory, and neurodegenerative disorders.^52^ Mechanisms of epigenetics modulation by environmental contaminants include alteration of DNA methylation, histone modification, and non-coding RNA (ncRNA) expression.^50^ The most investigated epigenetic mechanism is DNA methylation in gene promoters, acting as a repressor of gene expression.^52^ PAHs can affect regulatory T cell function through an epigenetic mechanism (methylation of FOXP3), increasing manifestations of atopic conditions such as asthma, conjunctivitis, and rhinitis.^53^ Indoor and outdoor exposition to PM_2.5_ has been associated with DNA methylation changes in children's nasal epithelial cells.^54^^,^^55^ ncRNAs contribute to many pathological and physiological processes, being one the main epigenetic markers in environmental exposure studies. MicroRNAs (miRNAs) are one of the three types of ncRNAs, participating in the regulation of gene transcription, protein expression, and protein complex assembly, although they do not encode proteins themselves.^50^ Heavy metals and PAH in PM_2.5_ are major contributors to ncRNA effects. There is observational evidence of a positive association between the expression of miRNAs and PAH exposition in children.^56^

## Mitigation and adaptation measures

Climate change is exacerbating the frequency and intensity of wildfires worldwide. It is crucial to develop public policies to prevent uncontrolled wildfire events and to mitigate its health impacts. Fire management strategies must consider global warming, reinforce the regulation on fire activity in rural and forested areas, and increase the resilience of fire-sensitive ecosystems.^57^ It is critical to establish monitoring networks for fire, weather, and air quality conditions. Data is needed to map wildfire risks, identify vulnerable populations, and develop efficient early warning systems. Current scientific knowledge and technology are able to predict wildfire risks and extreme climate and weather events,^58^ giving evidence-based support for preventive measures. Preparedness of healthcare facilities in susceptible regions should be improved, organizing human resources and supplies to a possible influx of wildfire smoke-related illnesses.^59^ Preventive measures also include the promotion of social awareness and education about the effects of climate change on fire regimes and household preparedness against WFS.

During a wildfire event, mitigation measures should be taken to attenuate the exposure to WFS, especially in wildland-urban interface regions. Staying indoors with windows closed is recommended if the fire fronts are kilometers away. Fans, closed-loop air conditioning and filter-based air cleaners may also be used to circulate the air and alleviate air pollution indoors.^60^ Proper hydration is fundamental to mitigate the risks of respiratory diseases driven by WFS. Dehydration dries out the upper and lower airways, reducing cilia beat frequency, damaging epithelial cells, and amplifying respiratory droplets that carry inhaled contaminants deeper into the lungs.^61^ Civil defense must be ready to evacuate socioeconomic and environmentally vulnerable communities without resources to mitigate exposure to WFS. Local governments should provide school-based activity recommendations in case of a wildfire incident. Surgical masks and respirators can provide limited protection for children during wildfire events, with expected decreases of roughly 20 % and 80 % for surgical masks and N95 respirators, respectively. One of the often-raised concerns is that the use of masks may provide a false sense of security if they do not provide adequate filtration and protection.^9^ Concerning the mitigation measures during a wildfire event, it is important to consider the costs and benefits of each unique situation when making decisions on behalf of children.

## Conclusion and recommendations

Wildfires are increasing in frequency and intensity worldwide, escalating environmental health threats associated with smoke exposure. The health impacts of WFS are manifold and systemic, affecting the respiratory, cardiovascular, and neural systems. Children are particularly vulnerable to wildfires, considering their physiological, anatomical, and behavioral characteristics. Although still a minority, the number of studies on the association between WFS and the health of children and adolescents has increased in the last decade. Most studies in the literature focus on WFS short-term exposure, revealing the acute adverse impacts for children and adolescents. The most significant health concerns associated with WFS are respiratory morbidities (e.g., asthma) and birth outcomes (e.g., preterm birth and low birth weight). It is important to conduct further studies on WFS impacts specifically on children and adolescents. Pediatric exposures to WFS may be exacerbated in comparison to adults, because children have higher minute ventilation per kilogram of body weight, and therefore experience a higher dose of air pollution than adults.

Given the increasing trends in wildfire activity and human settlements near risk areas, it is crucial to adopt mitigation and adaptation strategies, aiming to minimize the adverse health impacts of wildfire emissions. Preventive measures should be taken to avoid wildfire spread, including regulation and control of fire activity in rural areas; prediction of fire risk as a function of weather conditions; promotion of social awareness and education about the effects of climate change on fire regimes. During wildfire incidents, mitigation strategies should be undertaken especially in wildland-urban interface regions, including adequate response of the civil defense and health care system and recommendations for staying indoors.

## Conflicts of interest

The authors declare no conflicts of interest.
